# Unleashing the power of NK cells in anticancer immunotherapy

**DOI:** 10.1007/s00109-021-02120-z

**Published:** 2021-08-09

**Authors:** Meike Vogler, Senthan Shanmugalingam, Vinzenz Särchen, Lisa Marie Reindl, Victoria Grèze, Leon Buchinger, Michael Kühn, Evelyn Ullrich

**Affiliations:** 1 Institute for Experimental Cancer Research in Pediatrics, Goethe-University Frankfurt, Komturstrasse 3a, 60528 Frankfurt, Germany; 2 Children’s Hospital, Goethe-University Frankfurt, Frankfurt, Germany; 3 Experimental Immunology, Goethe-University Frankfurt, Frankfurt, Germany; 4 Department of Hematology, Medical Oncology, and Pulmonary Medicine, University Medical Center, Johannes Gutenberg-University, Mainz, Germany; 5 Frankfurt Cancer Institute, Goethe-University Frankfurt, Frankfurt, Germany

**Keywords:** Immunotherapy, Natural killer cells, Apoptosis, Cancer

## Abstract

Due to their physiological role in removing damaged cells, natural killer (NK) cells represent ideal candidates for cellular immunotherapy in the treatment of cancer. Thereby, the cytotoxicity of NK cells is regulated by signals on both, the NK cells as well as the targeted tumor cells, and the interplay and balance of these signals determine the killing capacity of NK cells. One promising avenue in cancer treatment is therefore the combination of NK cell therapy with agents that either help to increase the killing capacity of NK cells or sensitize tumor cells to an NK cell-mediated attack. In this mini-review, we present different strategies that can be explored to unleash the potential of NK cell immunotherapy. In particular, we summarize how modulation of apoptosis signaling within tumor cells can be exploited to sensitize tumor cells to NK cell-mediated cytotoxicity.

## NK cells for anticancer therapy

Among the players of the innate immune system, NK cells represent potent effectors which kill stressed and abnormal cells such as transformed malignant cells. NK cells are lymphocytes identified by the expression of an isoform of the neural cell adhesion molecule CD56 and by the concomitant absence of the CD3 antigen. According to their functional properties, NK cells can be classified into different subpopulations forming two main subsets of cytotoxic (CD56dimCD16 +) and immunoregulatory (CD56^high^CD16^dim/neg^) NK cells (reviewed in [1]).

NK cells can distinguish tumor cells from normal cells by a balance of inhibitory and activating receptors. In brief, inhibitory receptors, such as the killer immunoglobulin-like receptors (KIR), recognize major histocompatibility complex (MHC) class I molecules which are highly expressed in normal cells and prevent immune attack. Conversely, tumor cells often down-modulate MHC class I expression which induces the engagement of activating receptors, such as natural cytotoxicity receptors (NCR) and NKG2D. These activating receptors recognize the absence of MHC class I molecules and bind to stress-induced molecules (i.e., MIC A/B, ULBP 1–2, RAE-1, hemagglutinin of influenza virus) expressed on the tumor cell surface [2]. Upon ligation of the activating receptors, the immunoregulatory NK cells release pro-inflammatory cytokines like interferon (IFN)-γ, whereas the cytotoxic NK cells directly attack the target cell [3].

Due to their intrinsic characteristics, NK cells represent a promising therapeutic option for cancer patients. The efficacy of NK cell-mediated immunotherapy depends on the optimization of the choice of the cell source, on the one hand, and of the induction of NK cell cytotoxicity, on the other hand [4]. The first clinically applied NK cells were autologous cells derived from the same patient. Due to limited tumor recognition, these showed an insufficient anti-cancer response in clinical trials [5]. The feasibility and safety of allogeneic NK cells, derived from peripheral blood, umbilical cord blood, or induced from human embryonic or pluripotent stem cells has been demonstrated for hematological and solid tumors [6–9]. Accordingly, allogeneic NK cells derived from a haploidentical donor can prevent leukemia relapse and improve the outcome following hematopoietic stem cell transplantation without concomitant graft *versus* host disease [9, 10]. Also, in non-transplantation settings, adoptively transferred allogeneic NK cells can exert a cytotoxic effect against leukemic and tumor cells (graft *versus* tumor/leukemia effect) [11].

As an alternative to primary cells, the Food and Drug Administration (FDA)-approved NK cell line NK-92, which can be expanded to high numbers under good manufacturing practice conditions, can be used [12, 13]. Despite the numerous advantages of using this cell line, such as the cells’ constitutive activity caused by the lack of almost all inhibitory receptors, and their “off-the-shelf” availability, the in vivo proliferative capacity is limited due to the need of irradiation prior to transfer.

There are different strategies to improve NK cell cytotoxicity ex vivo which have been reviewed elsewhere [14], most prominently cytokine treatment (such as interleukin (IL)-15, IL-2 or IL-12/IL-15/IL-18) [15] or the use of feeder cell lines like K562, RPMI8866 or EBV-LCL, genetically altered to express different combinations of cytokines such as a membrane-bound form of IL-15 (mbIL-15), IL-21 or NK-stimulatory molecules like 4-1BB ligand or OX40 [4, 16–19]. NK cells expanded in the presence of K562-mbIL15-4-1BBL cells yielded an increased expansion up to 1000-fold compared to K562 feeder cells alone [19]. Furthermore, bi- or trispecific engagers can be used to provide close proximity for the formation of the immunological synapse by binding the target cell and NK cell simultaneously [4, 12, 20]. Currently, several immune cell engagers are being evaluated in clinical trials targeting amongst others CD30, CD33, or HER2 for the treatment of hematological and solid tumors (NCT03214666, NCT04101331, NCT01221571, NCT02665650, NCT04143711).

One limitation of NK cell-based immunotherapy is that tumor cells often escape the immune attack and develop resistance to apoptosis. Therefore, pharmaceuticals that may sensitize tumors to the NK cell-mediated immune attack are of high clinical relevance. To this end, several drugs or antibodies are currently being evaluated in clinical trials which are summarized in Table 1.

**Table 1:** List of clinical trials with adoptive NK cells investigated in combination with other therapeutics

NCT number	NK cells	Combination	Malignancy	Phase	First posted (Year)	Status
NCT00376805	Allogeneic NK cells	Cyclophosphamide, fludarabine	Breast cancer	2	2006	Terminated
NCT00625729	Donor NK cells	Rituximab, fludarabine, cyclophosphamide	CLL, non-Hodgkin lymphoma	1	2008	Terminated
NCT00698009	Haploidentical NK cells	Cyclophosphamide, fludarabine	Neuroblastoma	2	2008	Terminated
NCT00941928	Haploidentical NK cells	Epratuzumab	ALL	2	2009	Terminated
NCT01593670	Donor NK cells	Decitabine, vorinostat	MDS	2	2012	Completed
NCT02316964	Donor NK cells	Decitabine	AML	1	2014	Completed
NCT02370017	NK cell enriched lymphocytes (ANKL)	Docetaxel	Non-small cell lung cancer	2	2015	Unknown
NCT02734524	Autologous NK cells	Taxol, carboplatin	Non-small cell lung cancer	2	2016	Unknown
NCT02843126	NK cells	Trastuzumab	Breast cancer	1 and 2	2016	Completed
NCT02843204	Allogeneic NK cells	Pembrolizumab	Multiple cancers	1 and 2	2016	Completed
NCT02845856	NK cells	Cetuximab	Non-small cell lung cancer	1 and 2	2016	Completed
NCT02857920	Allogeneic NK cells	Bevacizumab	Multiple cancers	1 and 2	2016	Completed
NCT03056339	CD19 CAR-NK cells	Fludarabine, cyclophosphamide	ALL, CLL, non-Hodgkin lymphoma	1 and 2	2017	Recruiting
NCT03366064	Haploidentical NK cells	Pemetrexed	Non-small cell lung cancer	1	2017	Completed
NCT03554889	Autologous NK cells	Nimotuzumab	Multiple cancers	1	2018	Unkown
NCT03841110	Allogeneic NK cells (FT500)	Nivolumab, pembrolizumab, atezolizumab, cyclophophamide, fludarabine	Multiple cancers	1	2019	Recruiting
NCT03937895	Allogeneic NK cells	Pembrolizumab	Biliary tract cancer	1 and 2	2019	Recruiting
NCT03941262	Autologous NK cells (SKN01)	Avelumab, pembrolizumab	Multiple cancers	1	2019	Recruiting
NCT03958097	Autologous NK cells	PD-1/PD-L1 antibody	Non-small cell lung cancer	2	2019	Unknown
NCT04220684	Haploidentical NK cells	Cytarabine, decitabine, fludarabine	AML, MDS	1	2020	Recruiting
NCT04290546	Donor NK cells	Ipilimumab	Head and neck cancer	1	2020	Recruiting
NCT04558931	Autologous NK cells	Isatuximab	MM	2	2020	Not yet recruiting
NCT04796675	CD19 CAR-NK cells	Fludarabine, cyclophosphamide	ALL, CLL, non-Hodgkin lymphoma	1	2021	Recruiting
NCT04847466	Irradiated PD-L1 CAR-NK cells	Pembrolizumab	Gastric or head and neck cancer	2	2021	Not yet recruiting
NCT04872634	Autologous NK cells (SKN01)	Gemcitabine, cetuximab	Non-small cell lung cancer	1 and 2	2021	Not yet recruiting

## Induction of apoptosis by NK cells

**Fig. 1:**
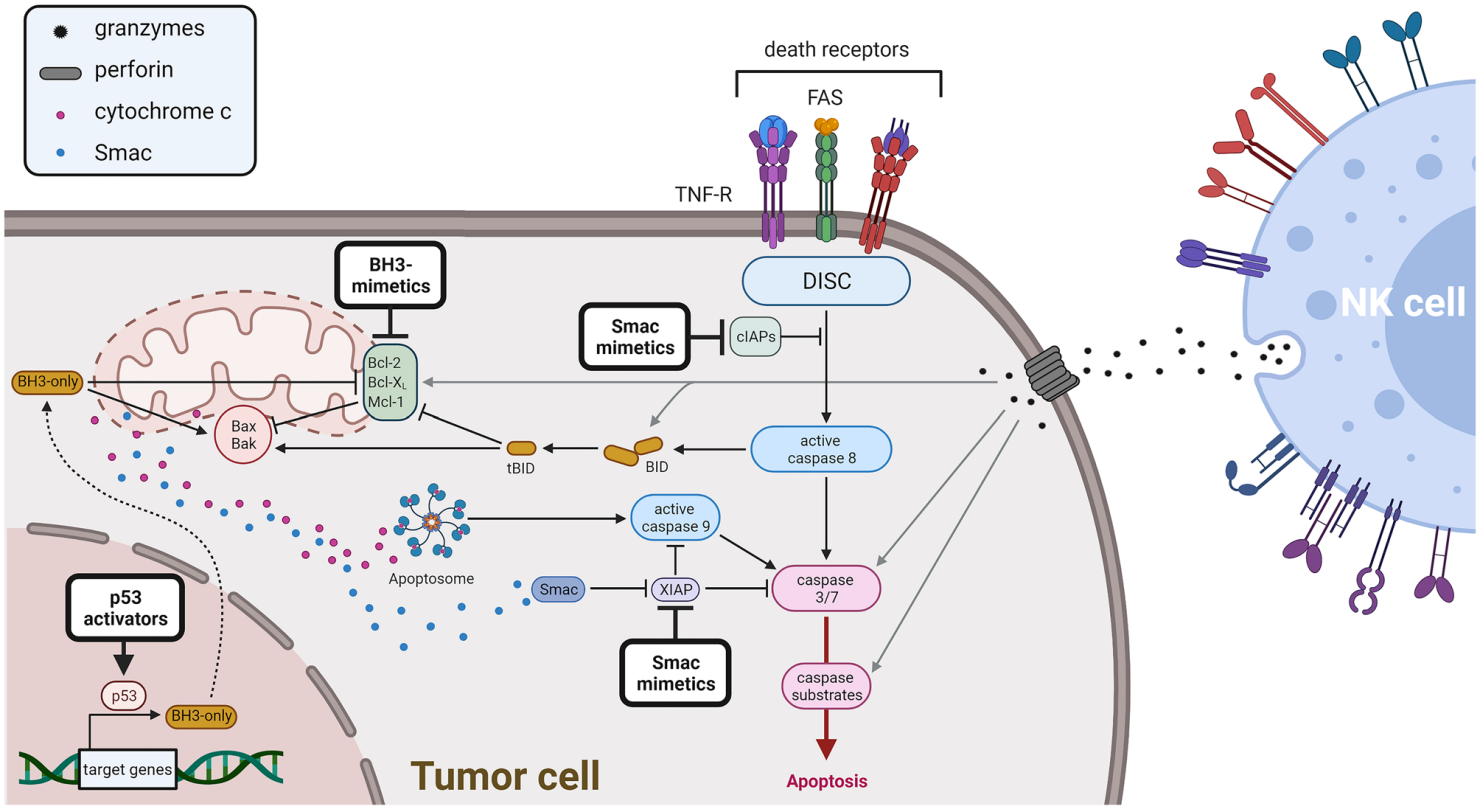
Proapoptotic therapeutics may potentiate NK cell-mediated cytotoxicity. Apoptosis can be initiated either at the mitochondria (intrinsic apoptosis) or upon ligation of death receptors on the plasma membrane (extrinsic apoptosis). This results in apoptosis signaling illustrated using black arrows. Apoptosis can be modulated using (a) Smac mimetics, (b) BH3 mimetics, (c) activators of p53, or (d) TRAIL agonists, thus overcoming apoptosis blockades within the tumor cell and facilitating NK cell-mediated attack, illustrated using gray arrows

Apoptosis can be initiated in the tumor cell via two separate but interconnected pathways (Fig. 1). Firstly, in the extrinsic pathway, the ligation of death receptors on the cell surface can result in the formation of the death-inducing signaling complex (DISC) and the activation of caspase-8 as initiator caspase. Secondly, cellular stress can trigger the activation of the intrinsic or mitochondrial apoptosis pathway. Here, stress sensors signal to the B-cell lymphoma 2 (Bcl-2) proteins to trigger the release of mitochondrial cytochrome c into the cytosol. Once in the cytosol, cytochrome c facilitates the assembly of the apoptosome, in which the initiator caspase-9 is activated. Both caspase-8 and caspase-9 can activate caspase-3, thus starting the caspase cascade and the biochemical and morphological characteristics of apoptosis [21].

The direct cytotoxicity of NK cells occurs upon formation of an immunological synapse linking the NK cell with the targeted tumor cell. Here, NK cells can release cytotoxic granule constituents including perforin and granzymes [22]. Granzymes are serine proteases that are specifically found in the cytotoxic granules of lymphocytes. Both granzyme A and granzyme B are expressed in NK cells, and upon perforin-mediated plasma membrane perforation, granzymes can enter the targeted cell to induce cell death by interacting with multiple players within the extrinsic and intrinsic apoptotic pathway.

Granzyme B has been shown to induce apoptosis by directly activating caspase-3, thereby initiating a mitochondrial amplification loop [23]. A similar study has determined that full activation of caspase-3 by granzyme B requires the release of pro-apoptotic factors from the mitochondria [24]. In addition, granzyme B can short-circuit the need for active caspase-8 as an amplification signal between the extrinsic and the intrinsic apoptotic pathway by cleaving the BH3-only protein BID to the active form tBID, thus directly promoting mitochondrial membrane permeabilization [25]. Further evidence indicating that mitochondria play an important role in granzyme B-mediated cell death has been provided by multiple studies showing that overexpression of the antiapoptotic protein Bcl-2 can prevent granzyme-mediated cell death [24, 26, 27]. Of note, granzyme B can also circumvent the need for active caspases and cleave the nuclear caspase substrate Inhibitor of caspase-activated DNase (ICAD), thus releasing caspase-activated DNase (CAD) and inducing DNA fragmentation independently of caspases [28, 29].

In a separate mode of action, NK cells can also induce extrinsic apoptosis using ligands like Fas ligand (FasL) or tumor necrosis factor (TNF)-related apoptosis inducing ligand (TRAIL), which are either expressed on the surface or secreted as a shorter soluble protein, and which can engage death receptors on the tumor cells. Death receptors belong to the TNF protein family and once engaged, these receptors oligomerize to facilitate DISC assembly. Assembly of the DISC leads to activation of caspase-8 as the apical caspase in the extrinsic apoptotic pathway. The two ways in which NK cells can kill tumor cells are not strictly separated, and in serial killing NK cells can switch from granzyme B-mediated killing to death receptor-mediated cytotoxicity [30].

## Sensitizing tumor cells to NK cell-mediated attack

Given that NK cells trigger apoptosis, alterations within the tumor cell that confer resistance to apoptosis may also build up a resistance to NK cell-mediated killing and allow immune escape. This however also implies that therapeutic strategies aiming to restore apoptosis sensitivity in cancer cells may be beneficial for NK cell-based immunotherapy. Hence, several molecularly targeted strategies may be applicable in combination with NK cells (Fig. 1).

### Smac mimetics

One of the mechanisms by which cancer cells acquire apoptosis resistance involves the inhibitor of apoptosis proteins (IAPs). Several IAPs are frequently overexpressed in cancer [31]. They function by inhibiting caspases in both the extrinsic and intrinsic apoptotic pathway. Their endogenous counter player is the mitochondrial protein second mitochondria-derived activator of caspases (Smac). Once the outer mitochondrial membrane is permeabilized during apoptosis, Smac is being released into the cytosol alongside of cytochrome c, and can inhibit IAPs, thus facilitating caspase activation. A first indication that the modulation of the balance between IAPs and Smac may increase the effects of immunotherapy has been obtained by overexpression of Smac in melanoma cells, which increased their sensitivity to granzyme B- or killer lymphocyte-mediated cytotoxicity [32]. As a therapeutic approach in cancer, apoptosis can be induced using small-molecule IAP inhibitors or Smac mimetics. We have recently shown that the Smac mimetic BV6 can improve NK cell-mediated killing of rhabdomyosarcoma cells, thus providing a proof-of-principle showing that an interference with the apoptotic regulation within the tumor cells can synergize with NK cell therapy [33]. Previously, Hodgkin lymphoma cell lines could be sensitized toward an NK cell-mediated killing by Smac mimetic treatment [34]. In a similar study, the Smac mimetic APG-1387 has been shown to increase NK cell-mediated killing of hepatocellular cancer in vitro and in vivo, indicating that this combination may be applicable and beneficial in a broad range of malignancies, possibly particularly in combination with immune-checkpoint inhibitors [35, 36].

### BH3 mimetics

By regulating the release of mitochondrial cytochrome c, the Bcl-2 proteins play a key role in the induction of apoptosis [37]. The Bcl-2 protein family contains antiapoptotic members, including Bcl-2, Bcl-X_L_, and Mcl-1, as well as proapoptotic proteins. Among the proapoptotic Bcl-2 proteins, there are the pore-forming proteins Bax and Bak, which are activated during apoptosis and facilitate the loss of mitochondrial membrane potential by integrating into the outer mitochondrial membrane. The other group of proapoptotic Bcl-2 proteins are the Bcl-2 homology domain 3 (BH3)-only proteins, which function as sensors for cellular stress and promote the activation of Bax and Bak. The antiapoptotic Bcl-2 proteins are frequently overexpressed in cancer and prevent apoptosis by blocking the activity of the proapoptotic Bcl-2 family members. Based on the nature of the interactions within the Bcl-2 protein family, the antiapoptotic Bcl-2 proteins serve as promising targets for the development of novel anticancer drugs [38–40]. With venetoclax/ABT-199, the first of these BH3-mimetics targeting Bcl-2 has been approved for the treatment of leukemia, highlighting the potential of the Bcl-2 proteins as targets in anticancer therapy [41]. Of note, in chronic lymphocytic leukemia (CLL), venetoclax is commonly administered to patients in combination with the anti-CD20 antibody rituximab, which increases antibody-dependent cellular cytotoxicity (ADCC) and improves the outcome compared to single treatment [42, 43]. Given that multiple studies have shown increased NK cell activation and cytotoxicity upon administration of rituximab [44–46], it is interesting to speculate that the combination of venetoclax with rituximab and NK cell immunotherapy may be advantageous in the treatment of cancer, in particular in B-cell malignancies. In support of the idea that targeting antiapoptotic Bcl-2 proteins with BH3 mimetics may increase NK cell-mediated cytotoxicity, a recent report has described that inhibition of Mcl-1, but not Bcl-2, increased the therapeutic efficiency of NK cells against acute myeloid leukemia (AML) cells [47].

### Activators of p53

The importance of the tumor suppressor gene *TP53* and its protein p53 in cancer has been reviewed extensively [47, 48]. Restoring the function of p53 is a well-studied avenue in cancer research, and several strategies to restore functional p53 in tumor cells have been identified [49]. Whereas some strategies involve the delivery of p53 in nanocomplexes, the small compound Nutlin-3a antagonizes the inhibitory interaction of MDM2 with p53, thus rescuing p53 function, albeit only in cells expressing wild-type p53. Among the many target genes of p53, there are several genes relevant for the induction of apoptosis, e.g., the BH3-only proteins Puma and Noxa, highlighting the potential of p53 restoration in the sensitization towards apoptotic stimuli. A recent study has shown that Nutlin-3a can increase surface expression of NKG2D ligands on neuroblastoma cells which coincides with increased cytotoxic activity of NK cells and reduced neuroblastoma growth in vivo, thus identifying a direct link between p53 function and NK cell activity [50]. Further support for a direct link between p53 and expression of NKG2D ligands has been provided by an earlier study showing p53-dependent expression of ULBP1/2 in cancer cells [51].

## Increasing the killing capacity of NK cells by modulation of the immune system

**Fig. 2:**
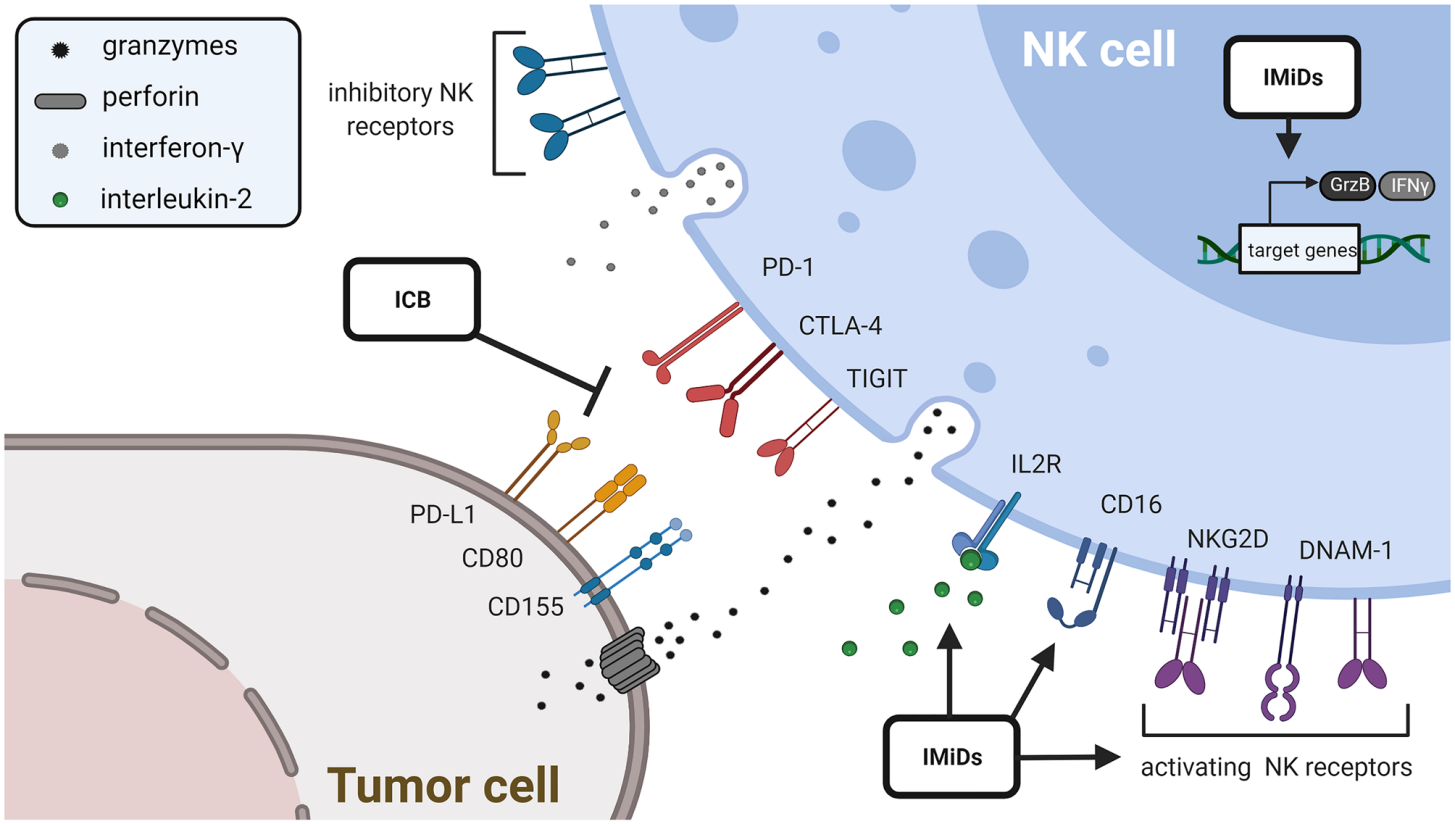
Modulation of the immune system using either IMiDs or ICB

As an alternative strategy, instead of sensitizing the tumor cells, the killing capacity of NK cells can be increased to facilitate better tumor cell killing. This can be achieved by modulation of the immune system using either immunomodulatory drugs (IMiDs) or immune checkpoint blockade (ICB) (Fig. 2).

### Effect of immunomodulatory drugs on the cytotoxicity of NK cells

The IMiDs thalidomide, lenalidomide, and pomalidomide have demonstrated antiangiogenic, immunomodulatory, and antiproliferative effects and are used especially for the treatment of multiple myeloma (MM) and myelodysplastic syndrome (MDS) with 5q deletion. In recent years, substantial progress toward discovering the mechanisms of action for IMiDs has been achieved. To this end, the cereblon receptor has been identified as a direct binding target of IMiDs. By binding to cereblon, an E3 ubiquitin ligase complex is formed leading to the selective degradation of transcription factors IKZF1 and IKZF2 in MM cells and CD1alpha in MDS with 5q deletion [79–81]. The discovery that IMiDs do not only bind to cereblon but also to the zeta-chain-associated protein kinase-70 (Zap-70) in T-cells and NK cells has provided further explanation of the enhanced antitumor toxicity of NK cells upon treatment with IMiDs [82].

The enhanced NK cell cytotoxicity upon administration of IMiDs can be attributed to indirect and direct effects on NK cells. Indirect effects include the increased IL-2 production of T-cells leading to an increase in NK cell cytotoxicity and ADCC in MM cells [83]. The elevated levels of IL-2 and the activation of NK cells are associated with the upregulation of CD69 on NK cells and increased IFN-γ synthesis [84]. In a severe combined immunodeficient (SCID) mouse model where T-cell function is impaired, the activation and recruitment of NK cells to tumor sites under treatment with lenalidomide seemed to be related to dendritic cells and their secreted cytokines [85]. Increased NK cell recognition and killing of MM cells has been reported by the indirect effect of increased expression of MICA and PVR/CD155 on MM cells which bind to the activating receptors NKG2D and DNAM-1 on NK cells [86]. For pomalidomide, an increase of the MHC class I expression on AML cells has been observed [87].

Direct activation of NK cell-mediated antitumor activity has been shown for lenalidomide, which increases expression of CD16, CD40L, and LFA1 on NK cells and thereby promotes ADCC [88]. In addition, upregulation of CD56 and downregulation of NKp30, NKp46, and KIR2D have been observed [87]. Moreover, IMiDs downregulate the suppressor of cytokine signaling 1 (SOCS1) in NK, NKT-, and T-cells [89]. An increased proportion of stimulated NK cells producing IFN-γ and a higher amount of IFN-γ produced per NK cell have been observed upon treatment with lenalidomide [90]. IMiDs can also enhance NK cell cytotoxicity via increased granzyme-B expression, which has been shown as a new direct effect on NK cells for pomalidomide and lenalidomide in a cereblon-mediated Zap-70-independent as well as a Zap-70-mediated cereblon-independent pathway [82]. It has further been reported that lenalidomide augments rearrangements in cortical actin at the immunological synapse of activated NK cells [90]. On the tumor side, IMiDs increase the polarization of lytic granules of AML cells in the NK cell immunological synapse [91].

Overall, the effects of IMiDs on NK cells have also been investigated in several clinical trials. Interestingly, MM patients with relapse treated with lenalidomide showed increased numbers of NKp44^+^ NK cells [92]. Under treatment with lenalidomide, patients with mantle cell lymphoma showed elevated levels of NK cells in relation to total lymphocyte counts [93]. To date, there are several other thalidomide analogues under development which show a higher affinity to cereblon, and the ongoing clinical trials will shed light on the effects of these new IMiDs on NK cell cytotoxicity and evaluate the most promising routes for combination treatments.

### Interactions between immune checkpoint blockade and NK cell therapy

In recent years, in particular, the field of immunomodulation with checkpoint inhibitors got international and interdisciplinary attention, which resulted in the award of the Nobel Prize to James P Allison and Tasuku Honjo in 2018. NK cells, still a niche within immunotherapy, also express immune checkpoint proteins, enabling the possibility for therapeutic intervention and enhancing NK cell-mediated cytotoxicity using ICB (summarized in Table 1).

### PD-1/PD-L1 blockade

Programmed cell death protein-1 (PD-1) is expressed on immune cells such as NK cells and other immune cells like activated T-cells, B-cells, and dendritic cells, whereas its ligand programmed death ligand 1 (PD-L1) is expressed on several types of tumor cells [94]. A high expression of PD-1 is found on NK cells in the peripheral blood, and the expression of PD-1 on NK cells is upregulated in cancer patients (summarized in [95]). Activated NK cells express PD-1 and, if it is engaged by PD-L1 expressed on the tumor cells, the NK cell-mediated tumor attack is suppressed. Consequently, PD-L1 expression in cancer cells resulted in reduced NK cell responses and generation of more aggressive tumors in vivo. Therefore, ICB is a promising therapy and blocking of the PD-1/PD-L1 axis caused a strong NK cell response in several mouse models of cancer which was indispensable for an efficient anti-cancer effect of ICB therapy [96].

In support of this, a recent study identified a synergistic NK cell-mediated cytotoxic effect together with anti PD-L1 treatment. Patient-derived ex vivo expanded NK cells successfully killed lung cancer cells, secreted IFN-γ thus re-activating immunosuppressed tumor infiltrated lymphocytes and increased PD-L1 expression on tumor cells, leading to a change in anti-PD-L1 response and an anti-PD-L1 second-hit strategy [97]. In triple-negative breast cancer, the anti-PD-L1-antibody avelumab triggered NK cell-mediated cytotoxicity and cytokine production. Avelumab significantly improved NK cell-mediated cytotoxicity against triple-negative breast cancer cells and tumor cells expressing higher levels of PD-L1 were shown to be more sensitive to avelumab-mediated ADCC. Also the stimulation of NK cells with IL-2 and IL-15 enhanced avelumab-triggered cytokine production and degranulation leading to an increased lytic activity against cancer cells [98].

First clinical evidence for a synergistic interaction between NK cells and ICB therapy has been provided by Lin and colleagues who showed in a clinical study that patients with advanced non-small cell lung cancer treated with pembrolizumab and allogenic NK cells had a longer median overall survival when compared with patients treated only with pembrolizumab (15.5 months vs. 13.3 months) [99].

To further increase the efficiency of ICB therapy, bispecific antibodies are currently being developed. To this end, enhanced efficacy in high-grade serous ovarian cancer has been shown for the bispecific antibody LY3434172, which simultaneously binds to PD-1 and PD-L1. LY3434172 has shown antitumor efficacy in vivo in humanized ovarian and other tumor xenograft mouse models. Of note, this bispecific antibody transits NK cells from inert to a more active and cytotoxic phenotype characterized by increased Myc and granzyme expression [100]. Even though monospecific ICB therapy has shown some clinical success, bispecific anti-PD-1/PD-L1-antibodies like LY3434172 are anticipated to be more effective thanks to their ability to engage two different receptors in the vicinity [101].

### Other checkpoint inhibitors

Apart from the classical PD-1/PD-L1 axis, NK cells express a broad variety of additional immune checkpoints. Cytotoxic T-lymphocyte-antigen 4 (CTLA-4), inhibiting the co-stimulatory signal of CD28, is a major target for ICB therapy. Inhibition of CTLA-4 using ipilimumab or tremelimumab successfully increased survival in melanoma patients [102, 103]. Compared to T-cells, the functional role of CTLA-4 in NK cells is not yet well understood. It has been shown that ipilimumab markedly increased killing and secretion of IL-2 and IFN-γ in NK cells [104]. In a more indirect manner, CTLA-4 targeting could deplete regulatory T-cells within the tumor site in a non-small cell lung cancer in vivo model, by that increasing the cytotoxic functionality of tumor-resident NK cells [105].

Recently, Deuse and colleagues have reported the SIRPα-CD47 axis, important in macrophage clearance and maturation of dendritic cells, as an inhibitory axis of NK cells. This inhibitory effect is dependent on a CD47 expression threshold on the target cells, orchestrating the killing kinetic. Furthermore, antibody-mediated blocking of CD47 on target cells could markedly increase cytotoxic effector function [106]. T-cell immunoreceptor with Ig and ITIM domains (TIGIT) as an additional inhibitory receptor expressed on T- and NK cells is getting more attention as a target for possible ICB therapy. Blockage of TIGIT by a novel antibody led to an increased NK cell-mediated cytotoxicity in an in vivo lung adenocarcinoma model [107]. A complementary approach is the reduction of CD155/PVR, the ligand of TIGIT. Treatment of lung adenocarcinoma cells with the natural product rediocide-A could decrease the expression of CD155/PVR and increase NK cell-mediated anti-tumor cytotoxicity [108]. Taken together, the combination of ICB therapy with allogenic NK cells may elicit increased anti-tumor activity and thus represents a promising new therapeutic approach which is currently being investigated in several early-phase clinical trials (Table 1).

## Modulating both NK cells and tumor cells

**Fig. 3:**
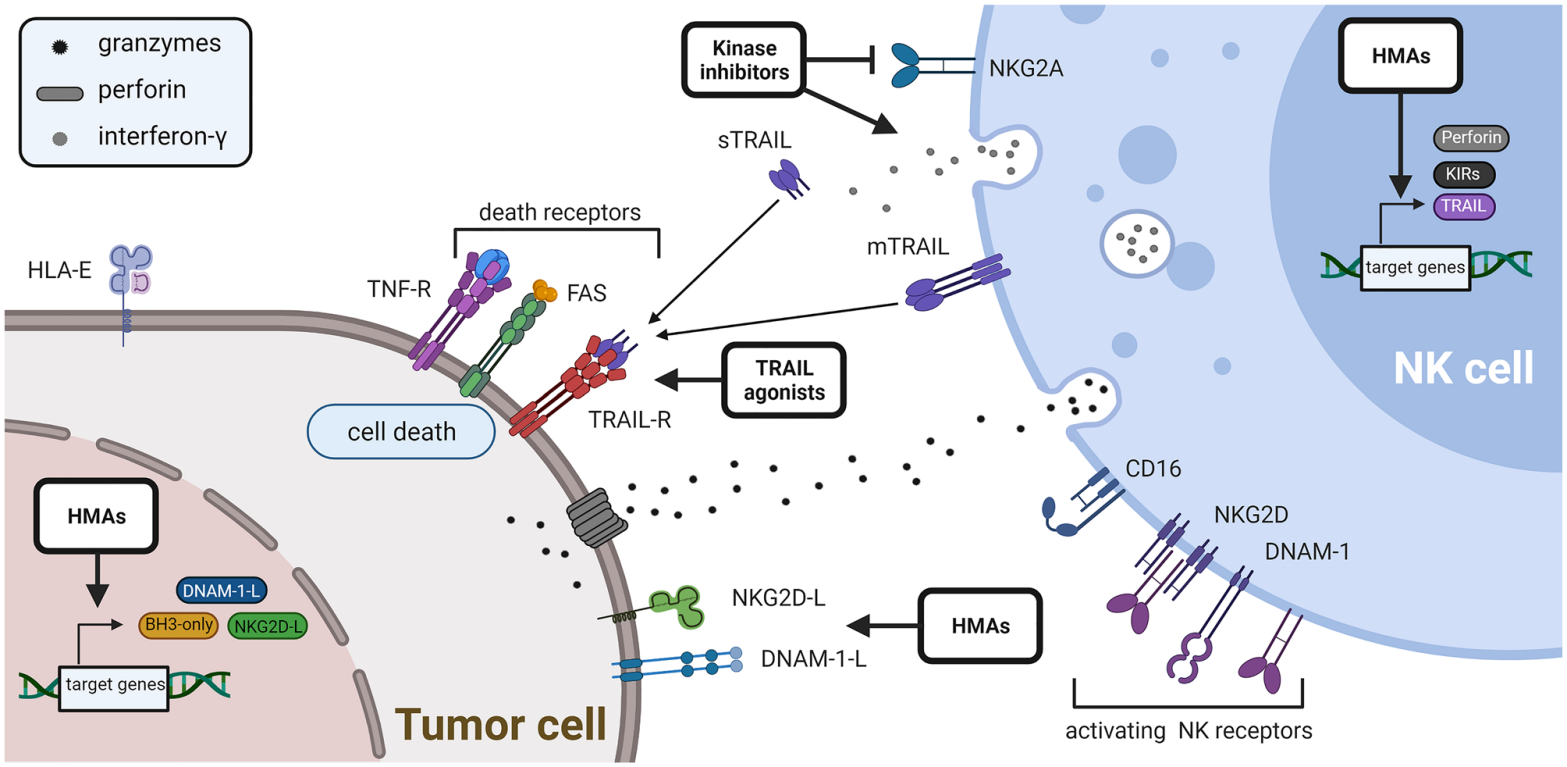
Effects of HMAs, TRAIL agonists, as well as kinase inhibitors

Several approaches have been identified that affect both NK cells and tumor cells, and thereby offer the opportunity to promote tumor cell killing from both sides. Here, we summarize the effects of hypomethylating agents (HMAs), TRAIL agonists, as well as kinase inhibitors (Fig. 3).

### Hypomethylating agents

As chromatin regulators are among the most commonly mutated genes in various human cancers and as epigenetic dysregulation has been implicated as an important mechanism of oncogenesis [59], drugs targeting epigenetic mechanisms have been introduced into clinical practice as promising cancer therapeutics (reviewed in [60]). One important example are first-generation epigenetic drugs such as the non-selective HMAs azacitidine and decitabine that are approved therapeutics for the treatment of high-risk MDS and AML [61]. While HMAs have been successfully combined with various immunotherapeutic approaches, these agents have been implicated as promising combination partners for NK cell adoptive transfer (reviewed in [62]). Recent preclinical studies demonstrated that low-dose HMA treatment did not impair viability, proliferation, or killing efficiency of NK cells but may cooperate with NK cells against AML by upregulating NKG2D- and DNAM-1-activating ligands in the malignant cells [63]. The upregulation of NKG2D by HMAs has recently been confirmed in one clinical trial [64]. In NK cells, decitabine has been reported to induce (re-)expression of TRAIL, perforin, and KIR receptors that are commonly silenced by promotor hypermethylation in these cells [63, 65]. It is interesting to note, that sensitization of AML cells to NK cell-mediated killing has been associated with the use of decitabine, whereas other studies reported that azacitidine exposure impaired NK cytotoxicity against AML cells [63, 66, 67]. While the definitive reasons for these heterogeneous results remain obscure, substantial differences in drug concentrations may explain at least some of the differences between these studies. Further insights will probably be provided by clinical trials (see also Table 1) that are currently assessing various therapeutic NK cell products in combination with HMAs for the treatment of AML (NCT02316964, NCT04220684).

Future studies will also explore a potential synergism between NK cell-based cancer immunotherapy and the large number of established and emerging second-generation epigenetic drugs that target specific chromatin modifications in selected cancer types [60].

### TRAIL agonists

TRAIL expression on the surface of cytotoxic NK cells is induced upon activation, e.g., by IL-2 or IL-15 [52]. Since its apoptosis-inducing receptors DR4 and DR5 are rarely expressed on healthy cells but frequently overexpressed on cancer cells, TRAIL-based therapies may be highly effective against cancer cells while sparing healthy cells [53]. Therefore, several strategies have been developed to trigger TRAIL-induced apoptosis, including the use of recombinant soluble TRAIL, agonistic DR4 or DR5 antibodies, or gene therapy approaches (reviewed in [54]). Despite promising results in preclinical research, the early clinical trials with both agonistic TRAIL receptor antibodies as well as recombinant TRAIL have been disappointing, probably due to suboptimal agonistic activity of the selected compounds and the occurrence of resistance mechanisms within cancer cells [55]. Currently, a new generation of TRAIL-based compounds that achieve high-order clustering and better agonistic activity are entering clinical trials, thus offering new hope for this approach [53]. Of note, some studies indicate that a combination of recombinant TRAIL together with an agonistic DR5 antibody induced higher-order clustering of the TRAIL receptor and thus increased proapoptotic effects [56, 57]. Given that TRAIL is expressed on NK cells, it may therefore be highly feasible to combine a DR5 agonistic antibody with NK cell immunotherapy. This has been shown in ovarian cancer, where the combination of the DR5 agonistic antibody AD5-10 sensitizes apoptosis-resistant cells to an NK cell-mediated killing in xenograft models, thus supporting the hypothesis that NK cell-mediated immunosurveillance may be relying on functional TRAIL signaling [57]. In neuroblastoma, expression of DR5 correlated with apoptosis induced by activated NK cells, and a TRAIL-blocking antibody reduced NK cytotoxicity in 14 out of 17 cell lines tested, demonstrating that TRAIL signaling contributed to NK cell-mediated killing. Notably, only membrane-bound TRAIL alone was responsible for apoptosis induction, whereas soluble secreted TRAIL did not contribute [58]. Taken together, these studies indicate that in particular the use of agonistic DR5 antibodies may overcome apoptosis resistance and synergize with TRAIL-mediated killing by NK cells.

### Immunomodulatory activity of kinase inhibitors

The inhibition of aberrant growth factor signaling using kinase inhibitors is a promising avenue to limit tumor growth and induce apoptosis, and therefore, several compounds are successfully being applied in the treatment of cancer. In addition to the direct effect on tumor cells, kinase inhibitors may also exhibit immunomodulatory abilities (reviewed in [68]). In chronic myeloid leukemia (CML), treatment with the tyrosine kinase inhibitor dasatinib increased NK proliferation and cytotoxicity [69–71]. Of note, long-lasting and complete responses after treatment of CML patients with dasatinib or imatinib are significantly associated with high NK cell proliferation in the blood [72, 73]. Mechanistically, dasatinib has been shown to reduce the expression of NKG2A on cells isolated from CML patients by inhibiting p38 mitogen-activated protein kinase (MAPK), thus altering the balance between inhibitory and activating receptors and increasing NK cell cytotoxicity [69]. In addition, gene polymorphisms of NKG2D were found to be associated with disease control by dasatinib [74].

Besides leukemia, kinase inhibitors have been shown to modulate immune responses in solid tumors. For example, MAPK and cyclin-dependent kinase 4/6 inhibitors act in concert to suppress the proliferation of KRAS-mutant lung cancer cells by triggering the accumulation of NK cells within the tumor and promoting NK cell-dependent tumor cell killing [75]. Also, the anti-tumor effects of the protein kinase inhibitor sorafenib have been linked with NK cell-mediated cytotoxicity. Accordingly, sorafenib can directly activate NK effector functions [76, 77] or induce a pro-inflammatory response in macrophages, which activates cytotoxic NK cells to attack hepatocellular cancer cells [78].

## Conclusion and outlook

Several approaches have demonstrated that the sensitization of tumor cells toward proapoptotic stimuli can improve NK cell-mediated tumor attack. However, it remains to be discovered which of these approaches will deliver clinical benefit and achieve long-lasting tumor remission without toxic side effects. Most likely, this will not be achieved by a “one type fits all” strategy but will have to be tailored to the tumor type and individual cancer characteristics. To further improve NK cell-based immunotherapy, it may also be an option to increase the killing capacity of ex vivo expanded NK cells. To this end, more efficient activation protocols have recently been established, such as the synergistic stimulation with IL-15 and IL-21 [109], and may be further developed. Alternatively, genetic engineering may be applied to artificially improve NK cell cytotoxicity, ideally specifically targeted against tumor cells (reviewed in [12]). This has been demonstrated using chimeric antigen receptor (CAR)-expressing NK cells (CAR-NK cells) (reviewed in [1]). Currently, most approaches are based on well-established targets adapted from CAR-T cells, including CD19 and CD22, and CAR-NK cells against these targets are being investigated in clinical trials for hematological malignancies (reviewed in [110]). In addition, early clinical trials are ongoing evaluating NKG2D ligands and several common tumor antigens as CARs expressed on NK cells (reviewed in [111]). These approaches may unleash the full potential of NK cells as immunotherapy, and results of the clinical studies are highly anticipated. In the future, combinations of apoptosis-sensitizing agents with CAR-NK cells specifically designed to target the individual tumor may be foreseeable and hold great potential to place NK cell immunotherapy at the frontline of cancer immunotherapy.
